# Advancing Functional Electrocatalysts for Hybrid Water Splitting: Strategies for Energy-Efficient Hydrogen Production

**DOI:** 10.3390/mi17050548

**Published:** 2026-04-29

**Authors:** Thirukumaran Periyasamy, Shakila Parveen Asrafali, Jaewoong Lee

**Affiliations:** Department of Fiber System Engineering, Yeungnam University, Gyeongsan 38541, Republic of Korea; thirukumaran@ynu.ac.kr (T.P.); shakilaparveen@yu.ac.kr (S.P.A.)

**Keywords:** electrocatalysts, functionalization, hybrid water splitting, sustainable hydrogen production

## Abstract

Electrocatalytic water splitting powered by renewable energy is a promising route for sustainable hydrogen production. Rather than developing separate catalysts for HER and OER, recent efforts focus on multifunctional electrocatalysts that can efficiently drive both reactions, simplifying system design and improving efficiency. A major limitation of conventional water splitting is the high overpotential and low-value oxygen production in OER. To overcome this, hybrid water splitting replaces OER with more valuable oxidation reactions, such as pollutant degradation or organic upgrading, enhancing overall energy and economic efficiency. This review covers the fundamentals of water splitting and highlights key physicochemical techniques for probing electrocatalyst activity, particularly structural reconstruction under operating conditions. It evaluates noble-metal, nonprecious-metal, and metal-free nanocarbon catalysts in both acidic and alkaline media, with emphasis on their roles in alternative anodic reactions. Finally, it outlines current challenges and future directions for developing efficient, durable, and sustainable electrocatalysts for advanced hydrogen production systems.

## 1. Introduction

As the global economy continues to expand rapidly and the population steadily increases, the demand for energy is reaching unprecedented levels. However, the reliance on nonrenewable fossil fuel reserves is becoming increasingly unsustainable, as these scarce resources are rapidly dwindling and will soon be inadequate to sustain humanity’s increasing energy demands. Additionally, the overconsumption of fossil fuels plays a major role in environmental damage by releasing toxic pollutants and greenhouse gases, which exacerbate climate change, air pollution, and ecological imbalances that pose serious threats to sustainable development. Given these pressing challenges, the search for alternative energy sources has become a global priority, making it crucial to create cost-effective, widely available, and eco-friendly energy solutions to lessen reliance on fossil fuels [1,2,3,4,5].

Among the promising alternatives, hydrogen is emerging as a highly efficient and clean energy source. Often referred to as the “cleanest energy in the world,” hydrogen has an exceptionally high calorific value and produces zero carbon emissions when used as fuel. Currently, hydrogen plays a vital role in several industries, including petrochemicals, ammonia production, metal refining, and fuel desulfurization. However, its potential applications extend far beyond these traditional sectors, with expectations that hydrogen will revolutionize transportation, aviation, electronics, maritime operations, and even the food industry in the coming years. The Hydrogen Council projects that global hydrogen production capacity will exceed 10 million tons by 2030, with demand potentially reaching 500 million tons annually by 2050. This substantial growth underscores hydrogen’s pivotal role in the global transition toward clean energy [3,4,5,6,7,8].

**Figure 1:**
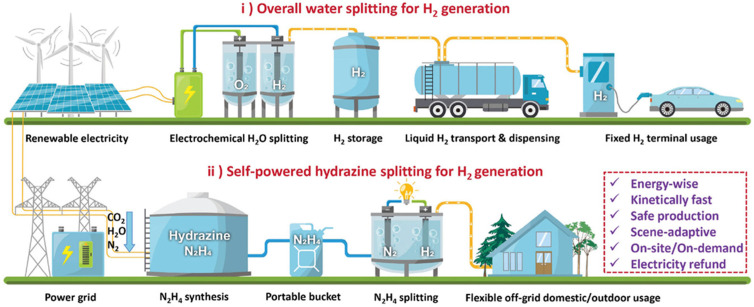
Workflow comparison of the overall water splitting and hydrazine splitting for H_2_ production and usage. Reprinted with permission from [4].

Despite its immense potential, the widespread adoption of hydrogen as a primary energy source faces significant challenges, particularly in production methods. Currently, most hydrogen is produced through steam methane reforming, a process that generates substantial carbon dioxide emissions and relies on nonrenewable natural gas. Alternative methods such as coal gasification and biomass conversion also produce considerable amounts of greenhouse gases, undermining the environmental benefits of hydrogen as a clean fuel. To truly harness hydrogen’s potential as a sustainable energy carrier, it is essential to develop production methods that are both environmentally friendly and economically viable. Among various hydrogen production technologies, water electrolysis stands out as one of the most promising approaches (Figure 1), as it uses electricity to split water into hydrogen and oxygen without generating harmful emissions when powered by renewable energy sources [1,9,10,11].

Water electrolysis involves two fundamental half-reactions occurring at separate electrodes immersed in an electrolyte solution. At the cathode, the hydrogen evolution reaction (HER) takes place, where water molecules or protons are reduced to produce hydrogen gas. Simultaneously, at the anode, the oxygen evolution reaction (OER) occurs, involving the oxidation of water or hydroxide ions to generate oxygen gas. The overall water splitting process requires a minimum thermodynamic potential of 1.23 V under standard conditions, but in practice, additional overpotentials are necessary to overcome kinetic barriers and drive the reactions at practical rates. These overpotentials are primarily determined by the efficiency of the electrocatalysts used at both electrodes, making catalyst development crucial for improving the overall efficiency of water electrolysis systems [12,13,14,15].

The efficiency of water electrolysis is significantly influenced by the choice of electrocatalysts, which facilitate the electrochemical reactions by lowering activation energy barriers. Noble metals such as platinum, iridium, and ruthenium have long been recognized as highly effective electrocatalysts due to their excellent catalytic activity and stability. Platinum-based catalysts exhibit exceptional performance for HER, while iridium and ruthenium oxides are considered state-of-the-art catalysts for OER. However, the widespread implementation of these noble metal catalysts is severely limited by their scarcity, high cost, and susceptibility to degradation under harsh operating conditions. These limitations have driven extensive research efforts toward developing alternative electrocatalysts based on earth-abundant transition metals, metal alloys, metal oxides, hydroxides, sulfides, phosphides, and carbon-based materials that can match or exceed the performance of noble metal catalysts while being more cost-effective and sustainable.

Recent advances in electrocatalyst design have led to the development of bifunctional catalysts capable of efficiently catalyzing both HER and OER, which simplifies system design and reduces costs by eliminating the need for separate catalysts at each electrode. Furthermore, researchers are exploring hybrid water splitting systems that replace the energy-intensive OER with thermodynamically more favorable oxidation reactions, such as the oxidation of small organic molecules, biomass derivatives, or waste pollutants [9,10,11,12,13,14]. These alternative anodic reactions not only reduce the overall voltage required for hydrogen production but also enable the simultaneous generation of valuable chemical products or environmental remediation, adding economic value and practical utility to the electrolysis process. This review provides a comprehensive overview of recent developments in functional electrocatalysts for both conventional and hybrid water splitting, examining their design principles, synthesis strategies, characterization methods, and performance evaluation in various electrolyte environments.

## 2. Fundamentals of Water Splitting

Water splitting is an electrochemical process that decomposes water molecules into hydrogen and oxygen gases through the application of electrical energy. This process occurs in an electrochemical cell consisting of two electrodes, an anode and a cathode, immersed in an electrolyte solution that facilitates ion transport between the electrodes. The overall water splitting reaction can be represented as 2H_2_O → 2H_2_ + O_2_, which requires a minimum thermodynamic potential of 1.23 V under standard conditions at 25 °C and 1 atm pressure. However, practical water electrolysis systems require significantly higher voltages, the thermoneutral voltage (1.48 V) (as it is directly related to heat balance during operation), due to various overpotentials associated with activation barriers, mass transport limitations, and ohmic resistances in the cell components [16,17,18,19].

The water splitting process involves two distinct half-reactions occurring simultaneously at the two electrodes. At the cathode, the hydrogen evolution reaction (HER) takes place, where electrons are transferred to water molecules or protons to produce hydrogen gas. At the anode, the oxygen evolution reaction (OER) occurs, involving the removal of electrons from water or hydroxide ions to generate oxygen gas. Both reactions proceed through multiple elementary steps involving the formation and breaking of chemical bonds, adsorption and desorption of intermediates on the catalyst surface, and electron transfer processes. The kinetics of these reactions are highly dependent on the nature of the electrocatalyst, the electrolyte composition and pH, temperature, and the applied potential.

### 2.1. Mechanism of Hydrogen Evolution Reaction (HER) Process

The hydrogen evolution reaction is a two-electron transfer process that can proceed through different mechanistic pathways depending on the electrolyte pH and the nature of the catalyst surface. In acidic media, HER typically begins with the Volmer step, in which a proton from the solution is adsorbed onto an active site on the catalyst surface and reduced by an electron to form adsorbed atomic hydrogen. This step can be represented as H^+^ + e^−^ + * → H*, where * denotes an active site on the catalyst surface. Following the Volmer step, hydrogen gas can be produced through either the Heyrovsky step or the Tafel step. In the Heyrovsky step, an adsorbed hydrogen atom reacts with another proton and electron from the solution to form hydrogen gas, represented as H* + H^+^ + e^−^ → H_2_ + *. Alternatively, in the Tafel step, two adjacent adsorbed hydrogen atoms combine to form hydrogen gas, represented as 2H* → H_2_ + 2*. The dominant pathway depends on the hydrogen binding energy of the catalyst surface and the reaction conditions [3,4,5,6,7].

In alkaline media, the HER mechanism is more complex due to the absence of free protons in solution, requiring water molecules to serve as the proton source. The Volmer step in alkaline conditions involves the dissociation of a water molecule at the catalyst surface, with one hydrogen atom being adsorbed and a hydroxide ion being released into the solution, represented as H_2_O + e^−^ + * → H* + OH^−^. This step is followed by either the alkaline Heyrovsky step, in which the adsorbed hydrogen reacts with another water molecule and electron to produce hydrogen gas and a hydroxide ion (H* + H_2_O + e^−^ → H_2_ + OH^−^ + *), or the Tafel step, which proceeds similarly to the acidic case. The additional water dissociation step in alkaline media generally results in slower HER kinetics compared to acidic conditions, making the development of efficient alkaline HER catalysts particularly challenging and important for practical applications in alkaline water electrolyzers.

### 2.2. Oxygen Evolution Reaction (OER) Process

**Figure 2:**
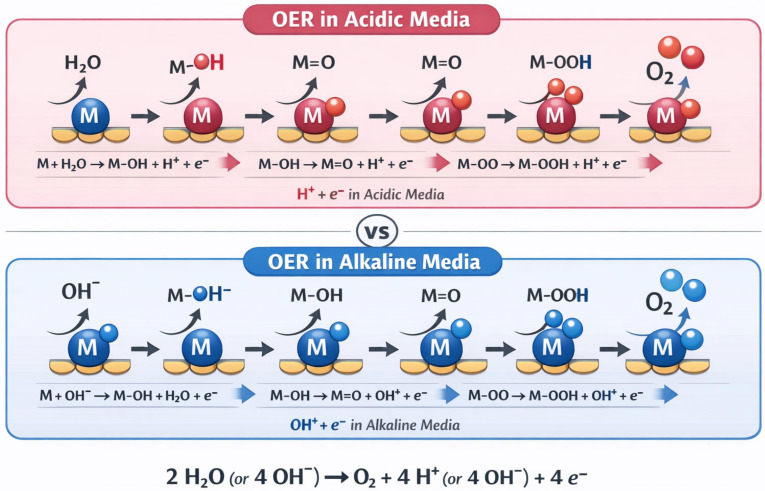
Schematic illustration of OER mechanism in acidic and alkaline medium.

The oxygen evolution reaction is a four-electron transfer process that is kinetically more sluggish than HER due to the involvement of multiple proton-coupled electron transfer steps and the formation of various oxygen-containing intermediates (Figure 2). In acidic media, the OER mechanism typically proceeds through a series of oxidation steps beginning with the adsorption of a water molecule on an active metal site (M) on the catalyst surface. The first step involves the oxidation of the adsorbed water molecule to form an adsorbed hydroxyl radical (M-OH) with the release of a proton and an electron. This is followed by further oxidation to form an adsorbed oxygen atom (M=O), then an adsorbed peroxide species (M-OOH), and finally the release of oxygen gas with the regeneration of the active site. Each of these steps involves the transfer of one electron and one proton, making the overall OER a complex four-electron process with multiple intermediates that must be stabilized on the catalyst surface [9,10,11,12,13].

In alkaline media, the OER mechanism follows a similar sequence of elementary steps but involves hydroxide ions as the reactant species instead of water molecules. The process begins with the adsorption of a hydroxide ion on the metal site, followed by sequential oxidation steps that form M-OH, M=O, and M-OOH intermediates before releasing oxygen gas. The formation and stabilization of these oxygen-containing intermediates are critical for efficient OER catalysis, and the optimal catalyst should have appropriate binding energies for each intermediate to facilitate the reaction without being too strong (which would block active sites) or too weak (which would prevent intermediate formation). The sluggish kinetics of OER, particularly the high overpotentials required to achieve practical current densities, represent one of the major bottlenecks in water splitting efficiency and have motivated extensive research into developing more active and stable OER catalysts.

### 2.3. Overall Water Splitting (OWS)

Overall water splitting refers to the complete electrochemical process in which both HER and OER occur simultaneously in a single electrochemical cell to produce hydrogen and oxygen gases. In a typical overall water splitting system, the same electrolyte is used for both half-reactions, and the system is designed to operate efficiently under identical conditions of pH, temperature, and electrolyte composition. The development of efficient overall water splitting systems requires careful consideration of several factors, including the selection of appropriate electrocatalysts for both electrodes, optimization of the electrolyte composition and concentration, management of gas evolution and bubble formation, and minimization of various losses including ohmic resistance, mass transport limitations, and electrode degradation.

One of the key challenges in overall water splitting is the need for catalysts that can function effectively under the same electrolyte conditions. While platinum-based catalysts excel at HER in acidic media and nickel-based catalysts perform well for OER in alkaline media, finding catalyst combinations that work optimally in the same electrolyte environment has been challenging. This has led to significant research interest in developing bifunctional catalysts that can catalyze both HER and OER with high efficiency, as well as in designing asymmetric electrolysis systems that use different electrolytes at the two electrodes separated by an ion-exchange membrane. Additionally, the practical implementation of overall water splitting systems must address issues such as product gas separation, system durability, scalability, and cost effectiveness to make the technology viable for large-scale hydrogen production applications [20,21,22,23].

## 3. Electrochemical Transformations of Catalysts: A Characterization Perspective

Understanding the structural and compositional changes that occur in electrocatalysts during operation is crucial for rational catalyst design and performance optimization. Electrocatalysts often undergo significant transformations when exposed to electrochemical conditions, including changes in oxidation state, surface restructuring, phase transitions, and the formation of new active species. These transformations can have profound effects on catalytic activity, selectivity, and stability, making it essential to characterize catalysts under operando or in situ conditions that closely mimic actual operating environments. Advanced characterization techniques have been developed to probe catalyst structure and composition during electrochemical reactions, providing valuable insights into the nature of active sites, reaction mechanisms, and degradation pathways.

### 3.1. Reconstruction Phenomena

Catalyst reconstruction refers to the structural and compositional changes that occur in electrode materials when subjected to electrochemical potentials, particularly during oxidation and reduction cycles. These reconstruction phenomena are ubiquitous in electrocatalysis and can involve various processes such as surface oxidation, hydroxylation, metal dissolution and redeposition, crystallographic phase transformations, and the formation of amorphous or disordered surface layers. Understanding reconstruction phenomena is critical because the active catalyst under operating conditions may be substantially different from the as-prepared material, and the true active sites responsible for catalytic activity may only form during the electrochemical activation process [20,21,22,23,24,25].

Metal and metal oxide catalysts are particularly prone to reconstruction under electrochemical conditions. For example, metallic nickel and cobalt surfaces readily oxidize to form hydroxides and oxyhydroxides when exposed to oxidizing potentials in aqueous electrolytes, and these oxidized species are often the true active phases for OER catalysis. Similarly, noble metal catalysts can undergo surface reconstruction involving changes in surface morphology, atomic arrangement, and coordination environment that significantly affect their catalytic properties. The extent and nature of reconstruction depend on various factors including the applied potential, electrolyte composition and pH, temperature, and the intrinsic properties of the catalyst material such as its composition, crystal structure, and defect density [13].

Recent studies have revealed that reconstruction can be beneficial or detrimental to catalytic performance depending on the specific system and reaction conditions. In some cases, electrochemical activation through controlled reconstruction can enhance catalyst activity by generating more active surface species, increasing the density of active sites, or improving electronic properties. In other cases, excessive reconstruction can lead to catalyst degradation through processes such as metal dissolution, structural collapse, or the formation of inactive phases. Therefore, understanding and controlling reconstruction phenomena through appropriate catalyst design and operating conditions is essential for developing high-performance and stable electrocatalysts for water splitting applications.

### 3.2. Electrochemical Characterization

Electrochemical characterization techniques provide fundamental information about catalyst activity, reaction kinetics, and electrochemical behavior under operating conditions. Cyclic voltammetry is one of the most widely used techniques for evaluating electrocatalyst performance, in which the potential is swept linearly between two values while measuring the resulting current. The cyclic voltammogram provides information about redox processes occurring at the catalyst surface, including the oxidation and reduction of the catalyst material itself and the onset potential for catalytic reactions. For water splitting catalysts, the key performance metrics extracted from cyclic voltammetry include the overpotential required to achieve a specific current density (typically 10 mA/cm^2^ as a benchmark), the current density at a given potential, and the shape of the polarization curve which reflects the catalytic kinetics. The oxidation behavior of Co species was examined by CV [11,12,13].

Linear sweep voltammetry (LSV) is similar to cyclic voltammetry but involves a single potential sweep in one direction, typically used to measure the polarization behavior of catalysts under steady-state or quasi-steady-state conditions. The Tafel slope, derived from the linear portion of the Tafel plot (overpotential versus log current density), provides important information about the reaction mechanism and the rate-determining step. Lower Tafel slopes indicate more favorable reaction kinetics and better catalyst performance. For HER, Tafel slopes of approximately 30, 40, and 120 mV/dec correspond to rate-determining steps of Tafel, Heyrovsky, and Volmer reactions, respectively, while for OER, the interpretation of Tafel slopes is more complex due to the multi-step nature of the reaction [19,20,21,22].

Electrochemical impedance spectroscopy (EIS) is a powerful technique for investigating the charge transfer kinetics and transport properties of electrochemical systems. In EIS measurements, a small sinusoidal potential perturbation is applied to the electrode over a range of frequencies, and the resulting current response is measured to determine the impedance as a function of frequency. The impedance data are typically analyzed using equivalent circuit models that represent different physical processes such as charge transfer resistance, double-layer capacitance, and mass transport limitations. For electrocatalysis, EIS can provide valuable information about the charge transfer resistance at the electrode-electrolyte interface, which is inversely related to the catalytic activity, as well as insights into the electrode porosity, electrolyte accessibility, and the presence of surface films or passivation layers.

The electrochemical surface area (ECSA) is an important parameter that quantifies the number of active sites available for catalysis and enables comparison of intrinsic activity between different catalysts. ECSA is typically determined by measuring the double-layer capacitance in the non-faradaic potential region, where no redox reactions occur, through cyclic voltammetry at different scan rates. The double-layer capacitance is proportional to the electrochemically accessible surface area, and by assuming a specific capacitance value for the material, the ECSA can be calculated. Normalizing catalytic currents by ECSA provides the specific activity, which reflects the intrinsic catalytic activity per unit surface area and is independent of the catalyst loading and electrode geometry. This normalization is crucial for meaningful comparison of different catalyst materials and for understanding structure–activity relationships [13,18,26,27].

Long-term stability is a critical requirement for practical electrocatalyst applications, and several electrochemical methods are used to assess catalyst durability under operating conditions. Chronopotentiometry involves applying a constant current density while monitoring the potential over time, with stable catalysts showing minimal potential increase during extended operation. Chronoamperometry applies a constant potential while measuring the current, with stable catalysts maintaining steady current output. Accelerated stability testing through repeated cyclic voltammetry scans between relevant potential limits can reveal degradation mechanisms and predict long-term performance. Post-stability characterization using various analytical techniques helps identify the structural and compositional changes responsible for performance degradation and provides insights for improving catalyst stability.

### 3.3. Spectroscopic Characterization

Spectroscopic techniques provide detailed information about the electronic structure, chemical composition, and molecular environment of electrocatalysts, complementing electrochemical measurements with atomic and molecular-level insights. Raman spectroscopy is particularly valuable for studying metal oxide and hydroxide catalysts, as it is sensitive to vibrational modes of metal-oxygen bonds and can distinguish between different phases and oxidation states [18].

X-ray photoelectron spectroscopy (XPS) is a surface-sensitive technique that provides quantitative information about elemental composition and chemical states of elements in the near-surface region of catalysts. XPS can detect changes in oxidation states, the presence of different chemical species, and surface contamination or modification. However, conventional XPS requires ultra-high vacuum conditions and cannot be performed during electrochemical operation, limiting its ability to capture the true state of the catalyst under working conditions. To address this limitation, ambient-pressure XPS and near-ambient-pressure XPS techniques have been developed that allow for measurements at higher pressures more representative of electrochemical environments, though still not in liquid electrolytes [22,24,25,26,27,28,29,30,31].

Ultraviolet-visible (UV-vis) spectroscopy is frequently used to analyze light absorption and electronic transitions in materials, providing insights into changes in oxidation states and electronic structure. Operando UV-vis spectroscopy can track fluctuations in metal oxidation states during electrochemical reactions in real-time. For example, studies on cobalt oxide catalysts have used UV-vis spectro-electrochemistry to monitor the oxidation of Co^2+^ to Co^3+^ and Co^4+^ species during OER, revealing that the concentration of oxidized species increases with applied potential and hydroxide concentration. The ability to correlate optical absorption features with specific oxidation states provides valuable information about the active species involved in catalysis and the potential-dependent evolution of catalyst composition [22,32,33,34,35].

X-ray absorption spectroscopy (XAS), including X-ray absorption near-edge structure (XANES) and extended X-ray absorption fine structure (EXAFS), provides element-specific information about local atomic structure, coordination environment, and oxidation states. XAS is particularly powerful for studying the structure of amorphous and highly disordered materials that are difficult to characterize by diffraction methods. Operando XAS measurements performed at synchrotron facilities have provided unprecedented insights into the structural evolution of electrocatalysts during operation, revealing phenomena such as metal dissolution, coordination changes, and the formation of catalytically active sites. The combination of XANES for electronic structure and oxidation state information with EXAFS for local coordination and bond distances provides a comprehensive picture of catalyst structure under working conditions [27].

### 3.4. Microscopic and Structural Characterization

Microscopic techniques provide direct visualization of catalyst morphology, structure, and composition at various length scales, from the macroscopic electrode level down to atomic resolution. Scanning electron microscopy (SEM) offers high-resolution imaging of catalyst morphology and surface features, revealing information about particle size and shape, surface roughness, porosity, and the uniformity of catalyst coatings on electrode substrates. Energy-dispersive X-ray spectroscopy (EDS) coupled with SEM enables elemental mapping and quantitative composition analysis with spatial resolution, which is valuable for characterizing multi-component catalysts and detecting elemental segregation or heterogeneity [33,34,35,36,37,38].

Transmission electron microscopy (TEM) provides higher resolution imaging than SEM and can resolve individual nanoparticles and crystalline domains. High-resolution TEM (HRTEM) can visualize atomic lattice fringes and crystallographic planes, enabling determination of crystal structures, lattice parameters, and the presence of defects such as dislocations, grain boundaries, and stacking faults. Selected area electron diffraction (SAED) and powder X-ray diffraction (XRD) provide complementary information about crystal structure and phase composition. Scanning transmission electron microscopy (STEM) combined with EDS or electron energy loss spectroscopy (EELS) enables atomic-resolution elemental mapping and electronic structure analysis, revealing the distribution of different elements in complex multi-component catalysts and at interfaces [33,34,35,36,37,38]. Advanced microscopy techniques such as aberration-corrected STEM and in situ/operando TEM are pushing the boundaries of what can be observed during electrochemical reactions. While operando TEM in liquid environments remains technically challenging, recent developments in liquid cell TEM holders and environmental TEM have enabled observation of catalyst behavior under conditions approaching those of electrochemical operation. These techniques have revealed dynamic processes such as nanoparticle coalescence, surface reconstruction, and the formation of amorphous surface layers that occur during catalyst activation and operation, providing direct visual evidence of the structural transformations that accompany changes in catalytic activity [39,40,41,42,43,44].

## 4. Electrocatalysts

### 4.1. Noble Metal-Based Electrocatalysts

Noble metals, particularly platinum, iridium, and ruthenium, have long been considered benchmark electrocatalysts for water splitting due to their exceptional catalytic activity and relatively good stability under electrochemical conditions. Platinum-based catalysts exhibit outstanding performance for HER across a wide pH range, with near-zero overpotentials and rapid reaction kinetics that approach the theoretical limits of catalytic efficiency. The exceptional HER activity of platinum is attributed to its optimal hydrogen binding energy, which allows for facile adsorption of hydrogen atoms without being so strong as to block active sites, satisfying the Sabatier principle for an ideal catalyst. However, platinum’s scarcity and high cost severely limit its use in large-scale water electrolysis systems, driving research toward reducing platinum loading through nano-structuring and alloying strategies.

Iridium and ruthenium oxides are recognized as the most active catalysts for OER in acidic media, which is particularly important for proton exchange membrane (PEM) water electrolyzers that operate under acidic conditions. These noble metal oxides exhibit relatively low overpotentials and good stability in acidic environments where most non-noble metal catalysts rapidly dissolve. The high activity of iridium and ruthenium oxides for OER is related to their ability to stabilize various oxygen-containing intermediates and facilitate multiple electron transfer steps. However, like platinum, the scarcity and high cost of iridium and ruthenium pose significant barriers to widespread commercialization, with iridium being one of the rarest elements in Earth’s crust.

Strategies to reduce noble metal usage while maintaining high catalytic performance include the development of nanostructured catalysts with high surface area, the synthesis of alloys and core–shell structures that maximize surface atom utilization, and the deposition of ultrathin noble metal layers on less expensive support materials. Nano-structuring approaches such as the synthesis of hollow nanoparticles, nanowires, nanosheets, and hierarchical porous structures can dramatically increase the surface-to-volume ratio, reducing the total amount of noble metal required while maintaining or even enhancing catalytic activity. Alloying noble metals with less expensive transition metals can not only reduce costs but also modify electronic properties and optimize binding energies for improved catalytic performance [40,41,42,43].

Core-shell nanostructures, in which a thin noble metal shell covers a core of less expensive material, represent an attractive approach to minimize noble metal usage while preserving high surface activity. The core material can be chosen to provide mechanical support, modify the electronic structure of the shell through strain or ligand effects, or contribute to catalytic activity in bifunctional systems. For example, platinum monolayer or few-layer shells on various core materials have demonstrated HER activity comparable to bulk platinum with drastically reduced platinum loading. Similarly, iridium-based core–shell structures have shown promising OER activity with significantly reduced iridium content compared to conventional catalysts.

Despite these advances in noble metal catalyst design, the fundamental limitations of scarcity and cost remain significant obstacles to large-scale deployment. This reality has motivated intensive research into developing alternative electrocatalysts based on earth-abundant elements that can match or approach the performance of noble metals while being economically viable for large-scale hydrogen production. The following sections discuss recent progress in non-precious metal catalysts and metal-free carbon-based materials that show promise for replacing or complementing noble metal catalysts in water splitting applications.

### 4.2. Transition Metal-Based Electrocatalysts

Transition metal-based electrocatalysts have emerged as promising alternatives to noble metals for water splitting applications, offering the advantages of natural abundance, lower cost, and tunable electronic and structural properties. First-row transition metals including iron, cobalt, nickel, copper, and manganese, along with their compounds such as oxides, hydroxides, sulfides, phosphides, nitrides, and carbides, have been extensively investigated as electrocatalysts for both HER and OER. These materials can achieve respectable catalytic performance through careful optimization of composition, structure, and morphology, although they generally require higher overpotentials than noble metal catalysts and may face challenges with stability, particularly in acidic media [38,39,40,41,42,43,44].

**Figure 3:**
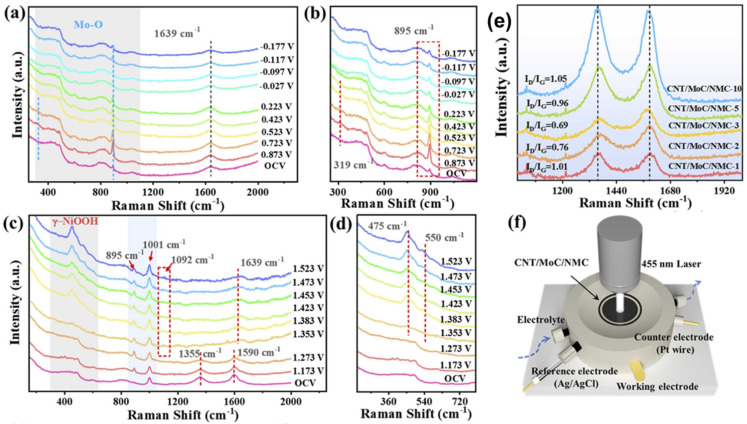
In situ Raman spectra of the CNT/MoC/NMC-3 as a function of applied potential vs. RHE in (**a**,**b**) HER; (**c**,**d**) UOR; (**e**) Raman spectra of the CNT/MoC/CoCH-x composites; and (**f**) Schematic illustration of the in situ Raman cell. Reprinted with permission from [18].

Nickel-based catalysts have received particular attention for alkaline water splitting due to their excellent OER activity in basic solutions and reasonable cost. Nickel hydroxides and oxyhydroxides are among the most active non-noble metal OER catalysts, with performance that can approach that of iridium oxide under alkaline conditions. The high OER activity of nickel-based materials is attributed to the formation of NiOOH species under oxidizing potentials, which serve as the active sites for oxygen evolution [44]. Liu et al. [18] used in situ Raman spectroscopy to probe surface states in the multicomponent heterostructure and monitored the intermediate evolution on the CNT/MoC/NMC-3 electrode. During HER, peaks at ~319 and 895 cm^−1^ appear at 0.873 V (vs. RHE), indicating oxidation of low-valent Mo to Mo^6+^–O species after activation (Figure 3a,b). With increasingly negative potentials, these peaks gradually weaken and disappear as hydrogen evolution dominates at −0.027 V, reflecting dynamic Mo restructuring via interaction between MoC and the CoNiMo alloy. For UOR, a peak at 1001 cm^−1^ (no bias) corresponds to the symmetric C–N stretch of adsorbed urea, while the 1092 cm^−1^ peak is assigned to CO_3_^2−^, a UOR product (Figure 3c,d). Ni activation during anodic oxidation is evidenced by the evolution of Ni-based intermediates, consistent with CV redox peaks associated with Ni^3+^ formation (Figure 3e).

**Figure 4:**
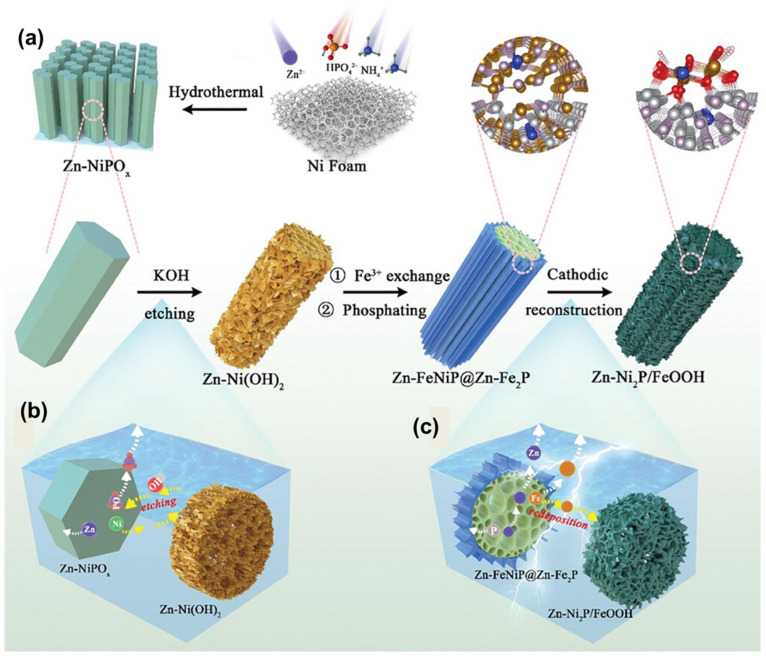
(**a**) Schematic illustration for the synthesis process of precatalyst and reconstructed Zn-Ni2P/FeOOH; (**b**) Schematic illustration of Zn dissolution facilitating the formation of multi-channel structures during KOH etching; and (**c**) Schematic diagram of self-optimization via a “dissolution-redeposition” dynamic mechanism during cathodic reconstruction. Reprinted with permission from [44].

**Figure 5:**
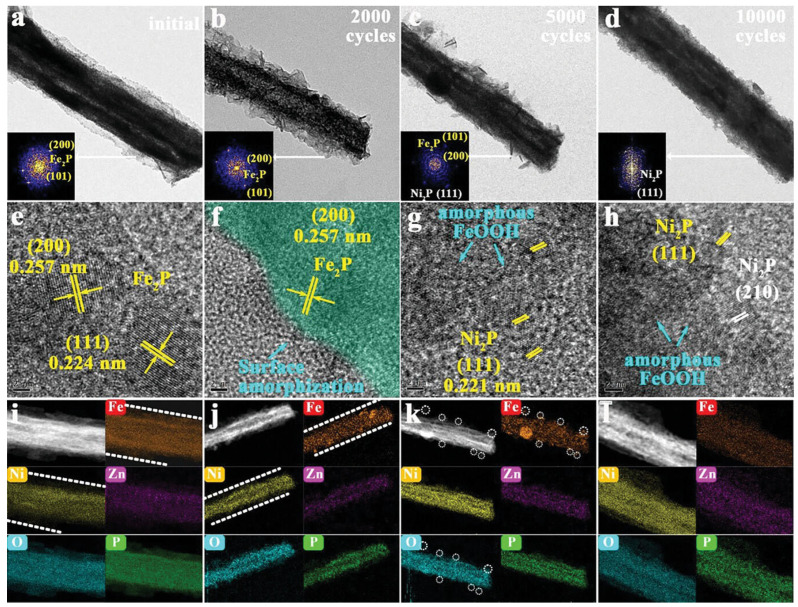
Structural evolution of reconstruction process during HER. (**a**–**d**) TEM, the insets show the corresponding fast Fourier transformation (FFT) images; (**e**–**h**) HRTEM and (**i**–**l**) HAADF-STEM images and the corresponding elemental mappings of Zn-FeNiP@Zn-Fe2P (initial) and CV-activated samples (after 2000, 5000, and 10,000 CVs (denoted as Zn-Ni2P/FeOOH) CV cycles). The white dotted lines in (**i**,**j**) represent the obviously observed reconstruction boundary between Fe2P and FeNiP. The white dotted circles in (**k**) represent the observed residual Fe2P after 5000 CVs activation during HER reconstruction. Reprinted with permission from [44].

Various strategies have been employed to enhance the activity of nickel-based catalysts, including the incorporation of iron as a dopant, which has been shown to significantly improve OER performance through electronic and structural modifications. Nickel-iron layered double hydroxides (LDH) represent a particularly promising class of OER catalysts that exhibit synergistic effects between the two metals (Figure 4). To elucidate the dissolution–redeposition mechanism, Jianhang Nie et al. [44] analyzed the microstructure of the Zn–FeNiP@Zn–Fe_2_P precatalyst during CV cycling (Figure 5a–d). TEM reveals a loosely porous surface that facilitates electrolyte infiltration and deep reconstruction. HRTEM (Figure 5f) shows surface amorphization, which becomes more pronounced after 5000 cycles, where thinner, sparser nanosheets form a loose porous structure (Figure 5c,g), with partial conversion of Fe_2_P into amorphous FeOOH. After 10,000 cycles, more Ni_2_P nanoparticles are exposed, generating abundant grain boundaries (Figure 5d) and crystalline/amorphous Ni_2_P/FeOOH interfaces (Figure 5h) due to continuous Fe_2_P dissolution, enhancing HER activity. EDS mappings (Figure 5i–l) further confirm the gradual decrease and dispersion of Fe and P signals during reconstruction.

**Figure 6:**
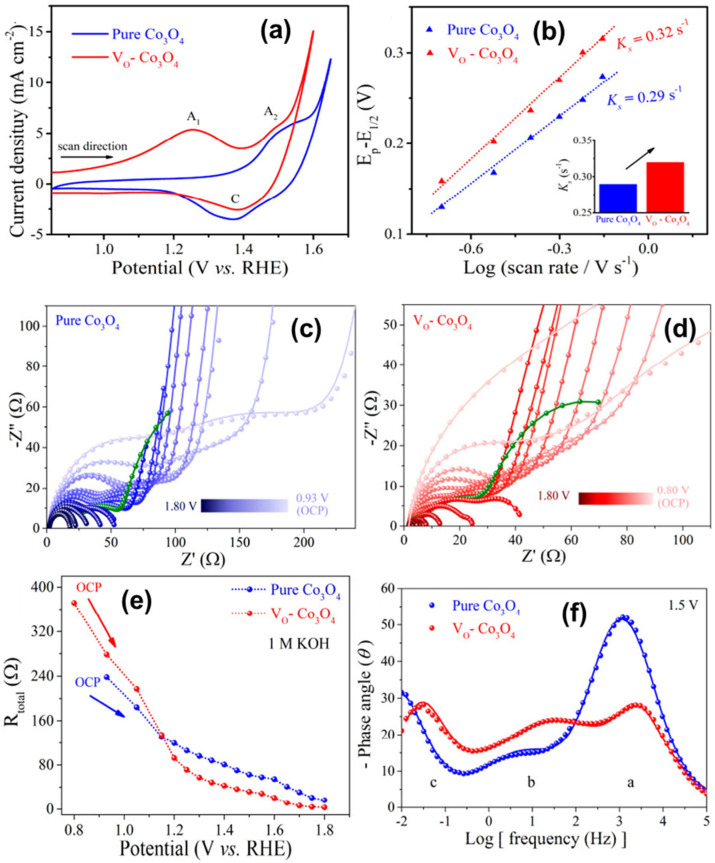
(**a**) First cyclic CVs measured for pure Co_3_O_4_ and VO−Co_3_O_4_ in 1 M KOH; (**b**) Ks of pure Co_3_O_4_ and VO−Co3O4; Nyquist plots for (**c**) pure Co_3_O_4_ and (**d**) VO−Co_3_O_4_ catalysts at different applied potentials versus RHE in 1 M KOH. The green data points indicated that the anode potential was 1.5 V; (**e**) response of the total charge transfer resistance (R_total_) to the applied potential of Co_3_O_4_ samples; and (**f**) Bode phase plots of pure Co_3_O_4_ and VO−Co_3_O_4_ at 1.5 V vs. RHE in 1 M KOH. Reprinted with permission from [13].

Cobalt-based materials, including cobalt oxides, hydroxides, phosphides, and sulfides, have also demonstrated excellent electrocatalytic activity for both HER and OER in alkaline media. Cobalt catalysts can undergo similar reconstruction phenomena to nickel-based materials, forming oxyhydroxide species under oxidizing conditions that are highly active for OER. As shown in Figure 6a,b, the anodic peak at 1.25 V (A1, vs. RHE) corresponds to the oxidation of Co^2+^ to Co^3+^, while the peaks at 1.47 V (A2) and 1.40 V (C) are assigned to the Co^3+^/Co^4+^ redox couple. The absence of a cathodic Co^3+^ → Co^2+^ peak indicates irreversible surface reconstruction of VO−Co_3_O_4_, forming a stable active surface for OER [11,12,13]. Operando EIS was employed to probe reaction kinetics and OH* adsorption at Co sites during OER. Nyquist plots (Figure 6c,d) show impedance evolution from OCP to 1.80 V in 1 M KOH. The parameters R_ct_ and CPE_ct_ represent OH^−^ adsorption resistance and pseudocapacitance, respectively, reflecting OH* evolution on the catalyst surface. VO−Co_3_O_4_ exhibits lower total resistance than Co_3_O_4_ above 1.15 V, indicating faster OH^−^ adsorption kinetics (Figure 6e). Bode phase plots at 1.5 V (Figure 6f) reveal two peaks: peak a (10^2^–10^4^ Hz) associated with Co–O_6_ (Oh) sites and peak b (10^0^–10^2^ Hz) with Co–O_4_ (Td) sites. Compared to Co_3_O_4_, VO−Co_3_O_4_ shows a positive shift in peak b and a reduced phase angle for peak a. An additional peak c appears at ≥1.50 V, attributed to accelerated deprotonation of Co–OOH* intermediates.

**Figure 7:**
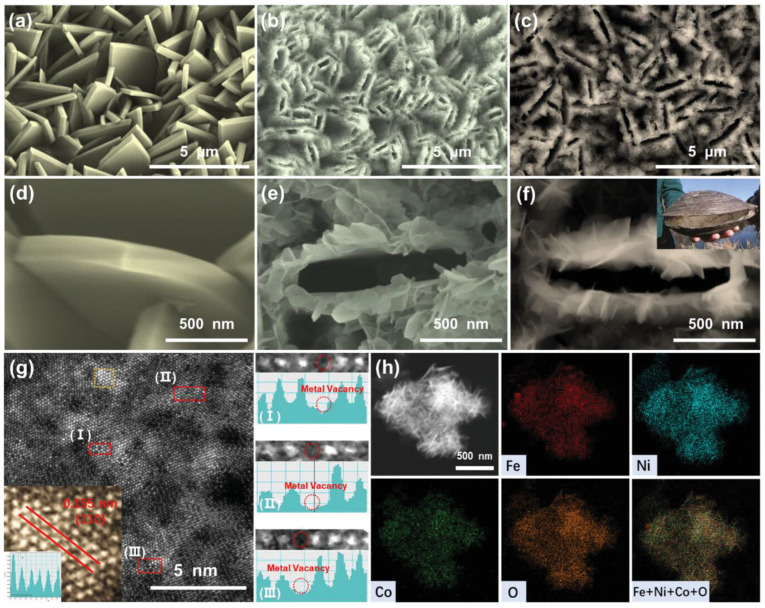
The SEM images of (**a**,**d**) Co-ZIF-L; (**b**,**e**) NiCo-LDH; and (**c**,**f**) Fe-NiCo-LDH. Inset in (**f**) is the optical photo of real mussel; (**g**) the AC-HAADFSTEM image of Fe-NiCo-LDH and corresponding atomic intensity profiles in three different regions, inset in (**g**) are the enlarged lattice diagram and contrast intensity profile along the red line; (**h**) the HAADF-STEM elemental mapping results of Fe-NiCo-LDH. Reprinted with permission from [38].

**Figure 8:**
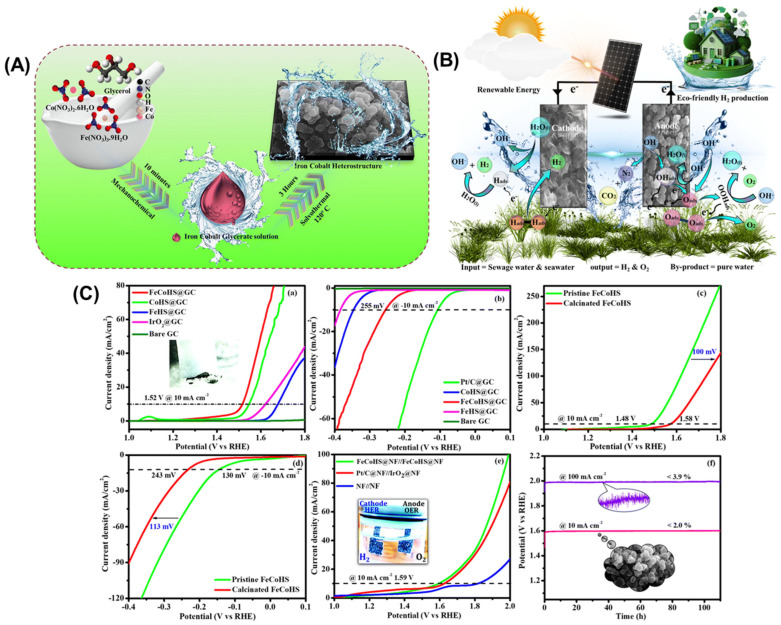
(**A**) Synthetic route of FeCoHS@NF. (**B**) Schematic of solar-assisted water splitting. (**C**) OER-HER activity comparison: (**a**) LSV showing OER of different electrocatalyst; (**b**) LSV showing HER of different electrocatalyst; (**c**) OER activity of pristine and calcinated FeCoHS@NF; (**d**) HER activity of pristine and calcinated FeCoHS@NF; (**e**) overall water splitting; and (**f**) stability in KOH. Reprinted with permission from [51].

Zhang et al. [38] used ZIF-based precursor with smooth leaf-like nanosheet arrays (Co-ZIF-L, Figure 7a,d) as a self-sacrificial template to derive LDHs, enabling preservation of its micro/nano morphology and porosity. SEM images show that both NiCo-LDH and Fe–NiCo-LDH form hierarchical, mussel-like hollow nanoarrays composed of interconnected ultrathin nanosheets (Figure 7b,c,e,f). Surface grooves arise from proton etching of Co-ZIF-L, while rapid outward diffusion of Co^2+^/Co^3+^ induces Kirkendall cavitation within the nanosheet template. AC-HAADF-STEM (Figure 7g) reveals crystal structure evolution and defect modulation after Fe incorporation, and EDS confirms the uniform distribution of Fe, Ni, Co, and O (Figure 7h). Mixed metal cobalt compounds, such as cobalt-iron and cobalt-nickel oxides, often exhibit enhanced performance compared to single-metal systems due to synergistic electronic effects and optimized binding energies for reaction intermediates. Cobalt phosphides have emerged as particularly promising HER catalysts in alkaline media, with some materials approaching the activity of platinum-based catalysts (Figure 8) [45,46,47,48,49,50,51].

**Figure 9:**
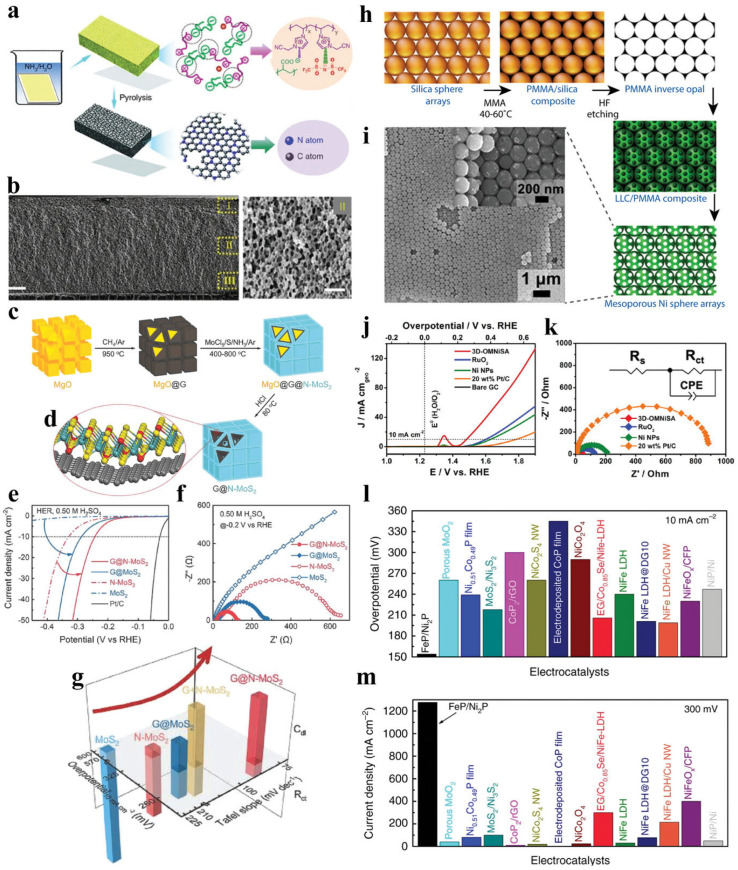
(**a**) Schematic diagram illustrating the preparation of hierarchically structured nitrogen-doped porous carbon membranes; (**b**) SEM image of the cross-section of HNDCM-250 000-1000 with scale bar of 20 μm (the high-magnification image on the right details the cross-sectional structure, with a scale bar of 500 nm); (**c**) synthesis process for 3D mesoporous G@N-MoS_2_ heterostructures using porous MgO templates; (**d**) structural model of G@N-MoS_2_, illustrating the van der Waals heterostructure with nitrogen doping and topological curvature [Color scheme: C (gray), Mo (green), S (yellow), N (red)]; (**e**–**g**) electrocatalytic HER performance of 3D mesoporous G@N-MoS_2_ heterostructures; (**h**) synthesis route; (**i**) SEM image; (**j**) OER; and (**k**) EIS of 3D ordered mesoporous nickel sphere; and (**l**,**m**) comparison of HER and OER overpotentials for bifunctional FeP/Ni2P hybrid catalyst. Reprinted with permission from [58].

Molybdenum-based materials, particularly molybdenum sulfides and molybdenum carbides, have attracted significant interest as HER catalysts due to their layered structures and favorable hydrogen binding properties. Molybdenum disulfide (MoS_2_) has been extensively studied as a potential replacement for platinum in HER applications (Figure 9), with the edge sites of MoS_2_ identified as the active centers for hydrogen evolution. Various strategies to increase the density of edge sites and improve conductivity, such as exfoliation into few-layer nanosheets, creation of sulfur vacancies, and hybridization with conductive carbon materials, have been pursued to enhance the HER activity of MoS_2_. Molybdenum carbides also show promise as HER catalysts due to their platinum-like electronic properties and good stability [52,53,54,55,56,57,58].

**Figure 10:**
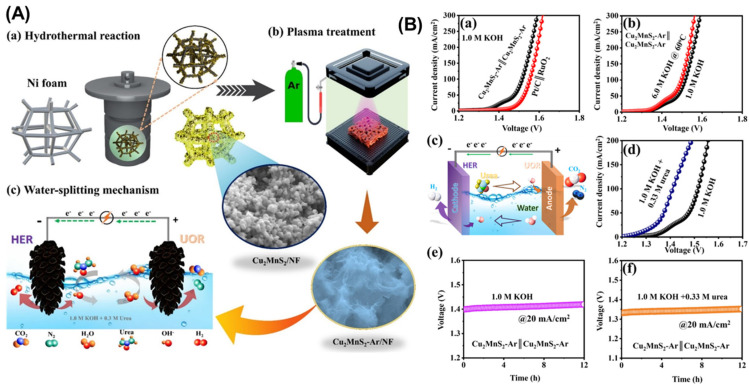
(**A**) A schematic illustration showing (**a**) the development of the Cu_2_MnS_2_ catalyst through a hydrothermal approach for electrochemical water splitting application; (**b**) surface modification in the Cu_2_MnS_2_ electrocatalyst through treatment using a plasma reactor employing Ar gas-based dielectric barrier discharge (DBD); and (**c**) a portrayal of a typical urea electrolytic cell showcasing its linked urea oxidation reaction (UOR) at the anode and hydrogen evolution reaction (HER) at the cathode. (**B**) Comparative analysis of (**a**) Pt/C||RuO_2_ and Cu_2_MnS_2_-Ar∥Cu_2_MnS_2_-Ar electrodes using the 2-E configuration in a 1.0 M KOH electrolyte; (**b**) linear sweep voltammetry (LSV) curves illustrating the electrochemical performance of Cu_2_MnS_2_-Ar||Cu_2_MnS_2_-Ar electrocatalyst in 1.0 M KOH and 6.0 M KOH electrolytes, conducted at 60 °C under industrial conditions; (**c**) Schematic representation depicting the mechanism governing the electrocatalytic activity toward urea oxidation reaction (UOR) and hydrogen evolution reaction (HER) in a 1.0 M KOH + 0.3 M urea electrolyte; (**d**) Performance of Cu_2_MnS_2_-Ar||Cu_2_MnS_2_-Ar electrodes in 1.0 M KOH and 1.0 M KOH + 0.3 M urea electrolytes; (**e**) Long-term stability assessment plot demonstrating the performance of the electrocatalyst in a 1.0 M KOH solution; and (**f**) Chronoamperometric stability plot over a period of 12 h, evaluating the electrocatalyst’s stability under constant current conditions. Reprinted with permission from [63].

Copper-based catalysts have been investigated for both water splitting and hybrid water splitting applications (Figure 10). While pure copper materials generally show moderate activity for HER and OER, copper-based compounds and alloys with other transition metals can exhibit enhanced performance. Recent studies have demonstrated that copper-molybdenum bimetallic catalysts show excellent activity for both HER and alternative oxidation reactions such as methanol oxidation, making them attractive for hybrid water splitting systems. The synergistic interaction between copper and molybdenum enhances electron transfer kinetics and optimizes the binding of reaction intermediates, leading to improved overall performance [59,60,61,62].

One significant limitation of many transition metal-based catalysts is their poor stability in acidic media, where metal dissolution becomes a major degradation pathway. While these materials can perform well in alkaline electrolytes, the development of acid-stable non-noble metal catalysts remains a major challenge. Some progress has been made through the synthesis of more stable compounds such as metal phosphides, carbides, and nitrides, as well as through protective coating strategies, but achieving stability comparable to noble metal oxides in acidic environments remains an ongoing research goal. Additionally, the optimization of transition metal catalysts requires careful consideration of multiple factors including composition, crystal structure, morphology, defect density, and electronic properties, making rational design challenging but offering numerous opportunities for innovation [63].

### 4.3. Metal-Free Carbon-Based Electrocatalysts

**Figure 11:**
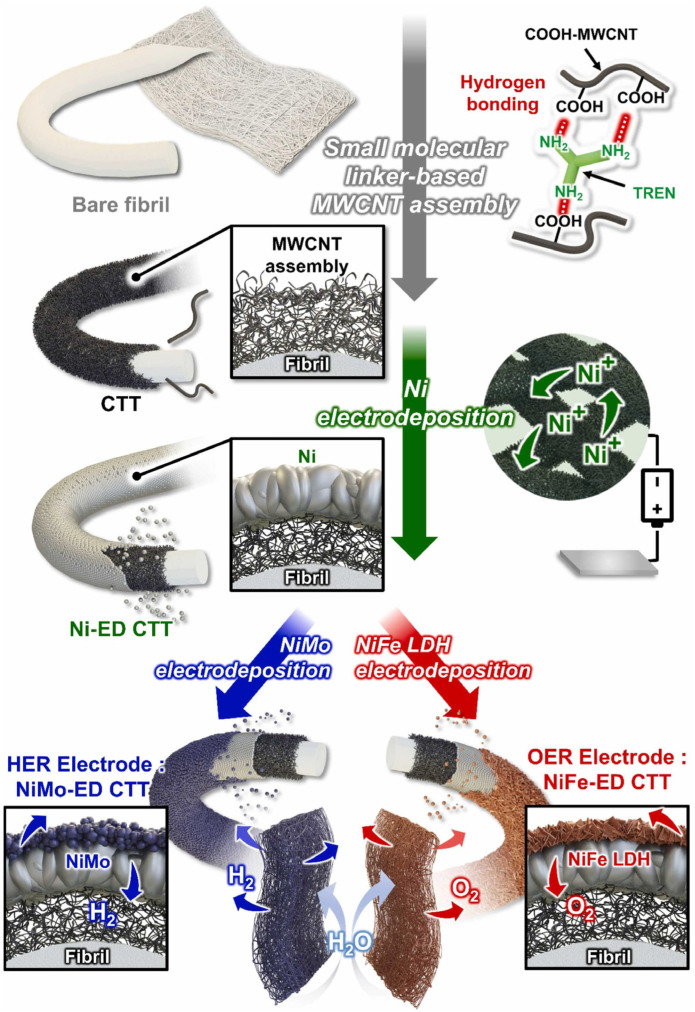
Schematic representation of the fabrication of water-splitting electrodes using electrodeposition induced by MWCNT/small molecular linker assembly. The structures of assembled electrodes were ideally represented. Reprinted with permission from [69].

Metal-free carbon-based materials have emerged as an exciting class of electrocatalysts that offer advantages including low cost, high abundance, excellent electrical conductivity, and good chemical stability. Various forms of carbon materials, including graphene, carbon nanotubes, carbon nitride, and heteroatom-doped carbons, have been investigated for water splitting applications (Figure 11). While pristine carbon materials generally show poor catalytic activity for HER and OER, their performance can be dramatically enhanced through heteroatom doping, defect engineering, and structural optimization. The development of highly active metal-free catalysts would represent a significant breakthrough for sustainable hydrogen production by eliminating dependence on scarce metal resources [64,65,66,67,68,69].

**Figure 12:**
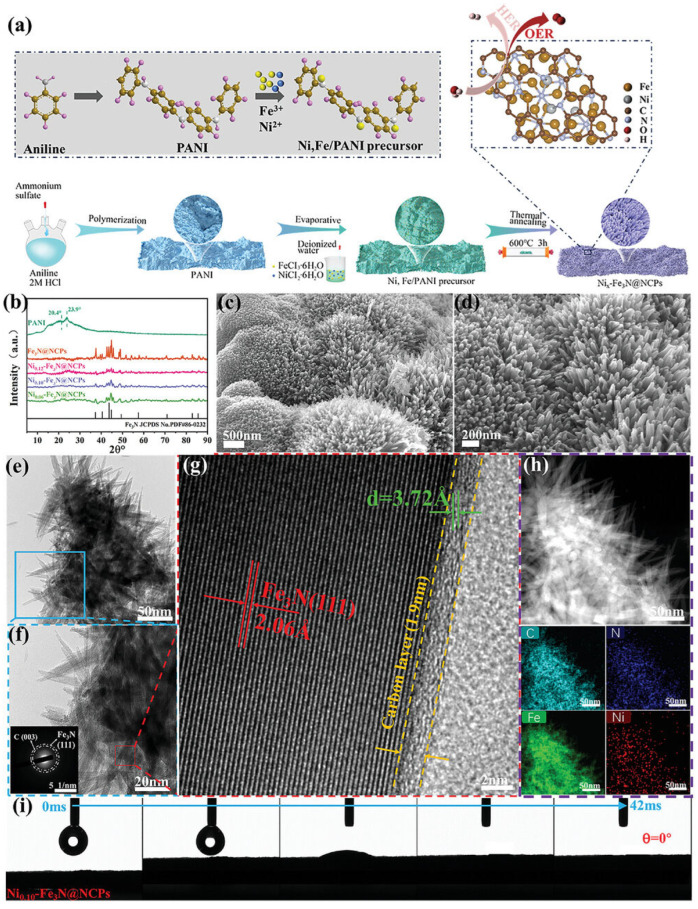
(**a**) Schematic illustration of the preparation process of Nix-Fe_3_N@NCPs samples; (**b**) XRD patterns; (**c**,**d**) SEM images of Ni_0.10_-Fe_3_N@NCPs; (**e**,**f**) TEM images and SAED pattern of Ni_0.10_-Fe_3_N@NCPs; (**g**) HRTEM image of Ni_0.10_-Fe_3_N@NCPs; (**h**) EDS elemental mapping images of C, N, Ni, Fe element of Ni_0.10_-Fe_3_N@NCPs; and (**i**) the hydrophilic contact angles of Ni_0.10_-Fe_3_N@NCPs. Reprinted with permission from [73].

**Figure 13:**
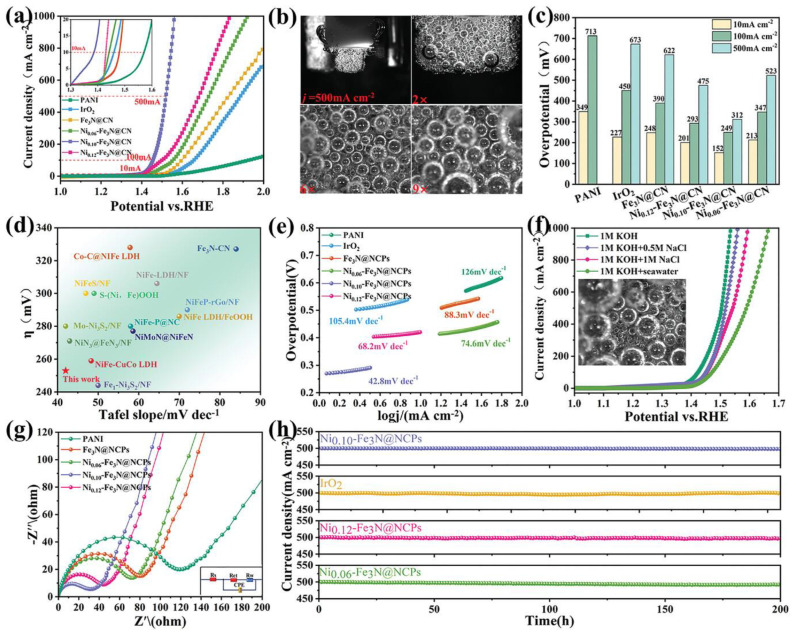
(**a**) OER Polarization curves of PANI, IrO_2_, Fe_3_N@NCPs, Ni_0.12_-Fe_3_N@NCPs, Ni_0.10_-Fe_3_N@NCPs, and Ni_0.06_-Fe_3_N@NCPs; (**b**) Optical image of the behavior of H_2_ bubbles on the electrode at a current density of 500 mA cm^−2^ during the OER processes; (**c**) Comparison of the overpotentials required to achieve the current densities of 10, 100, and 500 mA cm^−2^; (**d**) Comparing the overpotential and Tafel plots of Ni_0.10_-Fe_3_N@NCPs with other representative literatures; (**e**) The corresponding Tafel plots of PANI, Pt/C, Fe_3_N@NCPs, and Ni_x_-Fe_3_N@NCPs electrocatalyst; (**f**) Polarization curves of Ni_0.10_-Fe_3_N@NCPs in different electrolytes; (**g**) EIS Nyquist plots of PANI, Fe_3_N@NCPs, Ni_0.12_-Fe_3_N@NCPs, Ni_0.10_-Fe_3_N@NCPs, and Ni_0.06_-Fe_3_N@NCPs; and (**h**) Time-dependent current density curves of Ni_0.10_-Fe_3_N@NCPs, Pt/C, Ni_0.12_-Fe_3_N@NCPs, and Ni_0.06_-Fe_3_N@NCPs. Reprinted with permission from [73].

Heteroatom doping, particularly with nitrogen, phosphorus, sulfur, and boron, is the most common strategy for activating carbon materials for electrocatalysis. Nitrogen doping is especially effective, as nitrogen atoms can modify the electronic structure of carbon and create active sites for catalytic reactions (Figure 12 and Figure 13). Different nitrogen configurations, including pyridinic, pyrrolic, and graphitic nitrogen, have different effects on catalytic activity, with pyridinic nitrogen generally considered most beneficial for both HER and OER. The introduction of nitrogen atoms creates charge redistribution in the carbon framework, generating positively charged carbon atoms adjacent to nitrogen that can serve as active sites for oxygen reduction and evolution reactions.

Phosphorus and sulfur doping can also enhance the catalytic activity of carbon materials through similar electronic effects, and co-doping with multiple heteroatoms often produces synergistic effects that further improve performance. For example, nitrogen and phosphorus co-doped carbon materials have demonstrated excellent OER and ORR activity, approaching or even exceeding that of some metal-based catalysts in alkaline media. The optimization of dopant type, concentration, and configuration requires careful control of synthesis conditions, with various approaches including chemical vapor deposition, thermal treatment of heteroatom-containing precursors, and plasma treatment being employed to achieve desired doping levels and configurations [70,71,72,73].

Carbon nitride (C_3_N_4_) represents another promising class of metal-free catalysts with intrinsic nitrogen content and tunable electronic properties. Graphitic carbon nitride has a layered structure similar to graphite and contains abundant nitrogen atoms that can serve as active sites for catalysis. The band gap and electronic structure of carbon nitride can be modulated through various strategies including structural modification, defect introduction, and combination with other materials. While the catalytic activity of pristine carbon nitride is generally modest, significant improvements have been achieved through exfoliation into nanosheets to increase surface area, creation of porous structures to enhance mass transport, and hybridization with conductive carbon materials to improve electrical conductivity.

Despite the progress in metal-free carbon catalysts, their performance generally remains inferior to the best metal-based catalysts, particularly for OER which requires high overpotentials with carbon materials. The mechanisms of catalysis on heteroatom-doped carbons are still not fully understood, making rational design challenging. Additionally, the stability of carbon-based catalysts under harsh oxidizing conditions required for OER can be problematic, with carbon corrosion being a potential degradation pathway. Nevertheless, the continued development of metal-free catalysts represents an important research direction for sustainable electrocatalysis, and further advances in understanding structure–activity relationships and developing more active and stable carbon-based materials could lead to breakthroughs in this field [73].

## 5. Hybrid Water Splitting Systems

**Figure 14:**
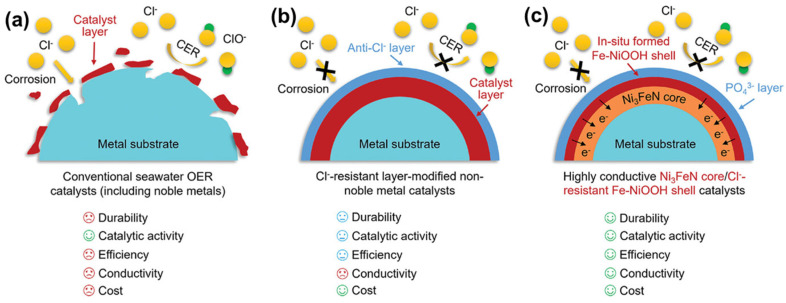
(**a**) Traditional OER catalysts (including noble metals) for seawater electrolysis are poisoned by Cl^−^; (**b**) Non-precious metal catalysts with anti-chlorine coatings resist Cl^−^ poisoning through a protective layer mechanism; and (**c**) The Ni_3_FeN core/PO_4_^3−^-modified Fe-NiOOH shell structure formed by in situ surface reconstruction of Ni_3_FeN@PO_4_^3−^, is designed for long-term seawater OER at industrial current densities. Reprinted with permission from [76].

Hybrid water splitting systems represent an innovative approach to hydrogen production that addresses the energy efficiency limitations of conventional water electrolysis by replacing the oxygen evolution reaction with thermodynamically more favorable oxidation reactions (Figure 14). The fundamental concept is based on the recognition that OER is kinetically sluggish and requires substantial overpotentials, typically 300–400 mV or more, to achieve practical current densities. By substituting OER with alternative anodic reactions that have lower oxidation potentials and faster kinetics, the overall cell voltage required for hydrogen production can be significantly reduced, leading to improved energy efficiency and lower electricity costs.

Various oxidation reactions have been explored as alternatives to OER in hybrid water splitting systems. Small organic molecules such as methanol, ethanol, glycerol, benzyl alcohol, and urea are attractive substrates because their oxidation typically requires 0.2–0.5 V lower potential than water oxidation. For example, methanol oxidation to formate or formic acid has a theoretical potential of only 0.03 V versus the reversible hydrogen electrode, compared to 1.23 V for water oxidation. This substantial reduction in anodic potential translates directly to lower cell voltages and improved energy efficiency. Additionally, the oxidation products of these organic substrates can be valuable chemicals, adding economic value to the hydrogen production process and creating opportunities for integrated chemical synthesis [74,75,76].

Methanol oxidation has been extensively studied as an alternative anodic reaction due to methanol’s high solubility in water, relatively simple molecular structure, and the value of its oxidation products. The complete oxidation of methanol to carbon dioxide involves six electrons, but partial oxidation to formaldehyde, formate, or formic acid, which are valuable chemical intermediates, can be achieved with high selectivity using appropriate catalysts. Transition metal-based catalysts, particularly those containing nickel, cobalt, and copper, have shown promising activity for methanol oxidation in alkaline media. The development of catalysts with high selectivity for partial oxidation products while maintaining good activity and stability represents an important research goal for practical methanol-assisted water splitting systems [77,78,79,80,81].

### 5.1. Nickel-Based Catalysts for Urea Oxidation

**Figure 15:**
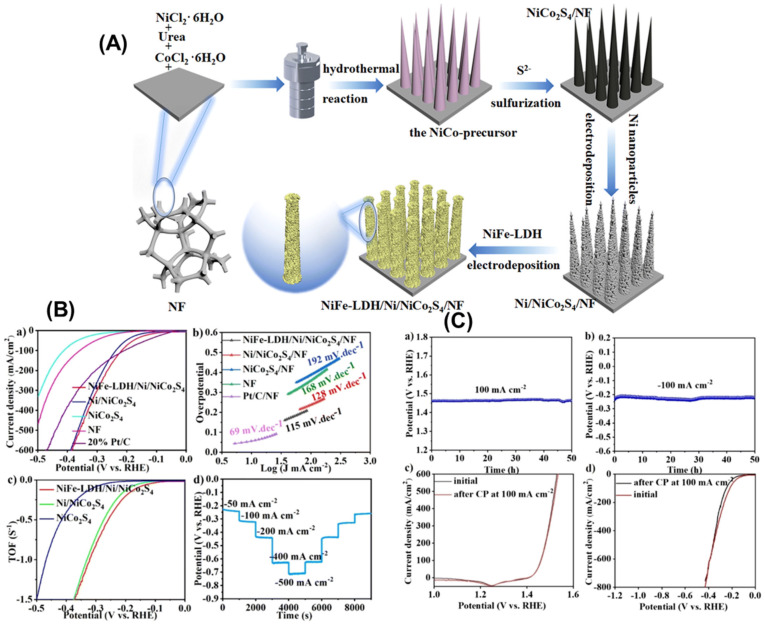
(**A**) Schematic diagram of the as-synthesized NiFe-LDH/Ni/NiCo_2_S_4_/NF electrode. (**B**) Electrochemical performance: (**a**) HER polarization curves; (**b**) Tafel slopes; (**c**) Calculation of TOF values; and (**d**) Potential–time curves at different current densities. (**C**) Stability tests: (**a**,**b**) OER and HER stability tests of NiFe-LDH/Ni/NiCo_2_S_4_/NF; (**c**,**d**) LSV curves before and after testing in OER and HER. Reprinted with permission from [81].

Urea oxidation has attracted particular interest for hybrid water splitting because urea is a major component of wastewater from agricultural and industrial sources, and its oxidation can simultaneously contribute to wastewater treatment and hydrogen production. The oxidation of urea in alkaline media has a theoretical potential of only 0.37 V, significantly lower than water oxidation, and produces nitrogen gas and carbon dioxide as products. Nickel-based catalysts are particularly effective for urea oxidation, with nickel oxyhydroxide species serving as the active phase (Figure 15). The integration of urea oxidation with hydrogen evolution in a hybrid electrolyzer not only reduces the voltage required for hydrogen production but also provides a sustainable approach to treating nitrogen-rich wastewater, addressing both energy and environmental challenges simultaneously [79,80,81].

Biomass derivatives such as glucose, furfural, and 5-hydroxymethylfurfural (HMF) represent another class of attractive substrates for hybrid water splitting. These compounds are derived from renewable biomass resources and their oxidation can produce valuable platform chemicals for the chemical industry. For example, the oxidation of HMF can produce 2,5-furandicarboxylic acid (FDCA), a bio-based monomer for polymer synthesis that can potentially replace petroleum-derived terephthalic acid. The integration of biomass valorization with hydrogen production creates synergies between renewable energy and sustainable chemistry, making the overall process more economically attractive and environmentally beneficial [82,83,84,85,86,87,88].

The practical implementation of hybrid water splitting systems requires careful consideration of several technical challenges. The catalyst must be highly active and selective for the desired oxidation reaction while being stable under operating conditions and resistant to poisoning by reaction products or impurities. The system design must ensure adequate mass transport of substrates to the anode surface and efficient removal of products to prevent accumulation and catalyst deactivation. Additionally, the compatibility of the hybrid system with existing electrolyzer technologies and the economic viability of the overall process including substrate costs and product values must be carefully evaluated. Despite these challenges, hybrid water splitting represents a promising direction for making hydrogen production more efficient and sustainable while creating opportunities for integrated chemical synthesis and waste treatment.

### 5.2. Copper-Molybdenum Capsule Catalyst for Hybrid Water Splitting

**Figure 16:**
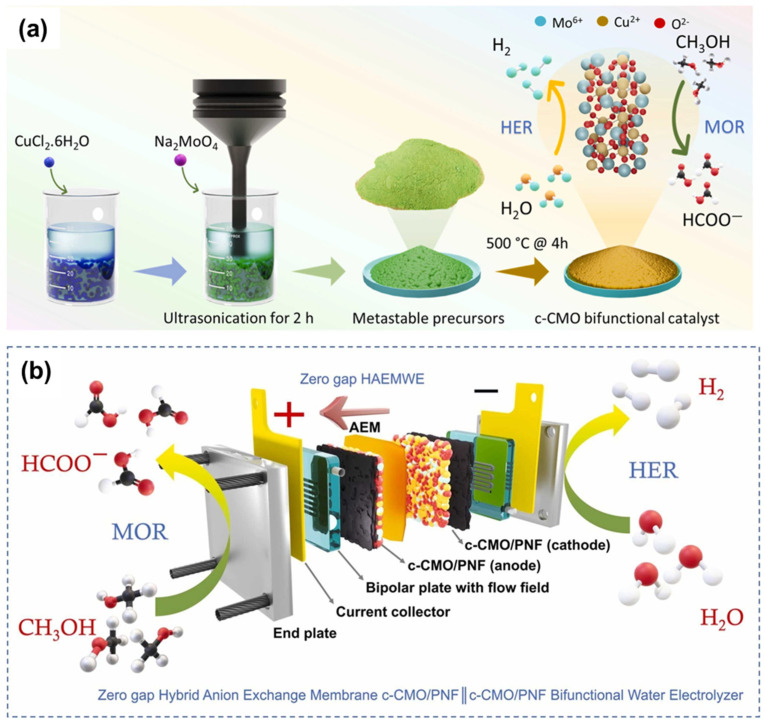
(**a**) Schematic illustration of the synthesis of c-CMO by ultrasonication-assisted sonochemical method and their application toward hybrid water electrolysis systems; and (**b**) Assembly of c-CMO/PNF‖c-CMO/PNF bifunctional water electrolyzer. Reprinted with permission from [27].

**Figure 17:**
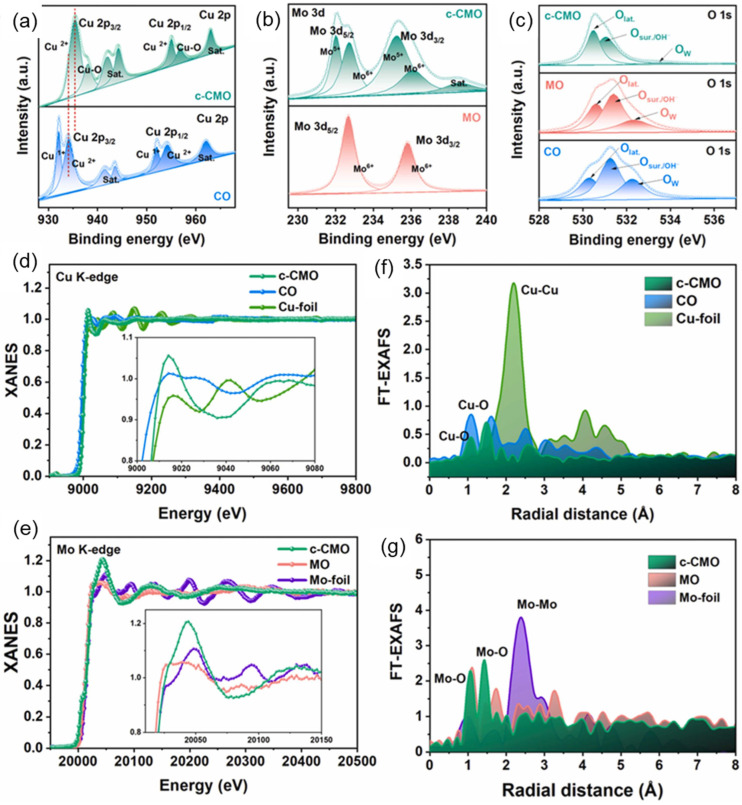
High-resolution XPS deconvolution spectra of (**a**) Cu 2p; (**b**) Mo 3d; and (**c**) O 1 s in CO, MO, and c-CMO catalysts, respectively; (**d**) normalized Cu K-edge XANES spectra of CO, c-CMO and Cu foil; (**e**) normalized Mo K-edge XANES spectra of spectra of MO, c-CMO and Mo foil; (**f**) Magnitudes of Fourier transform Cu K-edge EXAFS oscillations of CO, c-CMO and Cu foil; and (**g**) Magnitudes of Fourier transform Mo K-edge EXAFS oscillations of MO, c-CMO, and Mo foil, respectively. Reprinted with permission from [27].

A recent study demonstrated the development of a highly efficient capsule-like copper-molybdenum oxide (c-CMO) catalyst supported on porous nickel foam for application in hybrid anion exchange membrane water electrolyzers. The catalyst was synthesized through a two-step process involving the initial preparation of copper oxide nanorods on nickel foam followed by the deposition of molybdenum oxide to form a capsule-like core–shell structure, as shown in Figure 16. This unique morphology provides several advantages including high surface area, abundant active sites, and strong interaction between copper and molybdenum components that enhances catalytic activity and stability. As shown in Figure 17a, the Cu 2p spectra of CO and c-CMO exhibit characteristic Cu 2p_3_/_2_ and Cu 2p_1_/_2_ peaks with satellite features. The Mo 3d spectra (Figure 17b) display two main peaks corresponding to Mo 3d_5_/_2_ and Mo 3d_3_/_2_ states. The O 1s spectra (Figure 17c) can be deconvoluted into three components at ~530.4, 531.7, and 533.4 eV, assigned to lattice oxygen (O_lat_), surface/defect oxygen (O_sur_), and adsorbed water (O_w_), respectively. XANES analysis (Figure 17d,e) shows that the Cu K-edge of c-CMO shifts to higher energy with increased white-line intensity compared to Cu_2_O and Cu foil, indicating a higher Cu oxidation state. The Mo K-edge exhibits a pre-edge feature (~20,000 eV) attributed to s(Mo) → 4d(Mo) + 2p(O) transitions. FT-EXAFS (Figure 17f,g) reveals dual coordination shells: Cu–O bonds at ~1.73 Å and 2.3–2.7 Å, and Mo–O bonds at ~1.55 Å and ~1.8 Å, corresponding to MoO_6_ octahedra. These results confirm the distinct coordination environment and structure of c-CMO [27].

**Figure 18:**
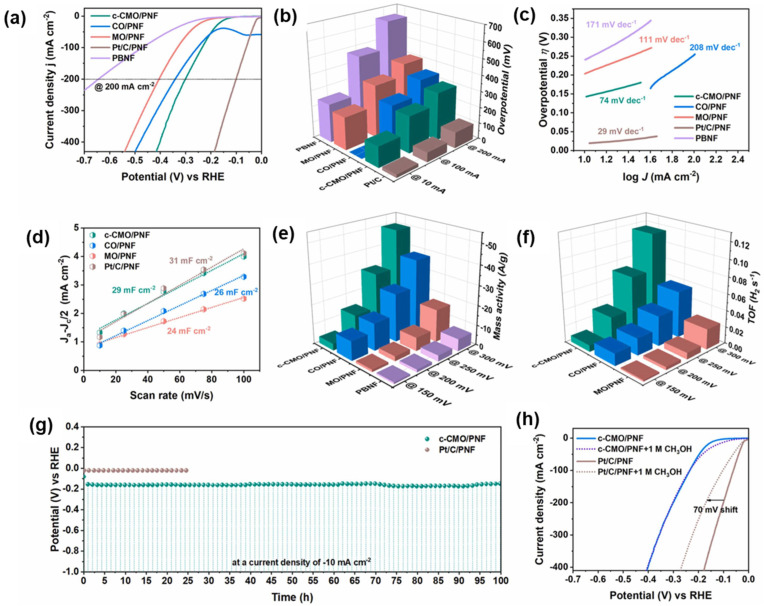
Electrochemical HER performances showing (**a**) Hydrogen evolution reaction profiles; (**b**) HER overpotential analysis; (**c**) corresponding Tafel analysis; (**d**) measured C_dl_; (**e**,**f**) calculated mass activity and turnover frequency plots; (**g**) The HER Stability test of c-CMO/PNF for 100 h and Pt/C/PNF electrode for 25 h in 1.0 M KOH electrolyte at -10 mA cm^−2^; and (**h**) Methanol tolerance test of c-CMO/PNF and Pt/C/PNF in addition of 1 M CH_3_OH. Reprinted with permission from [27].

The electrochemical characterization of the c-CMO catalyst revealed exceptional performance for both hydrogen evolution and methanol oxidation reactions in alkaline media (Figure 18). For hydrogen evolution, the catalyst exhibited an overpotential of only 130 mV at a current density of 10 mA/cm^2^, which is remarkably low for a non-noble metal catalyst and approaches the performance of commercial platinum catalysts. The Tafel slope of 74 mV/dec indicates favorable reaction kinetics following the Volmer-Heyrovsky mechanism. The electrochemical surface area calculation based on double-layer capacitance measurements showed that the c-CMO catalyst has an ECSA of 725 cm^2^, comparable to platinum catalysts, confirming that the capsule-like structure provides abundant accessible active sites for catalysis [27,28,29].

For methanol oxidation, the c-CMO catalyst demonstrated superior activity compared to individual copper oxide or molybdenum oxide catalysts, requiring only 1.39 V to achieve 10 mA/cm^2^, which is 0.239 V lower than the potential required for oxygen evolution. This substantial reduction in anodic potential directly translates to improved energy efficiency in the hybrid water splitting system. Nuclear magnetic resonance analysis revealed that the catalyst exhibits excellent selectivity for formate production with a Faradaic efficiency of approximately 96%, demonstrating that the methanol oxidation proceeds selectively to the desired partial oxidation product rather than complete oxidation to carbon dioxide.

The stability testing of the c-CMO catalyst showed excellent durability under continuous operation at 10 mA/cm^2^ for 100 h with minimal performance degradation. The catalyst also exhibited outstanding methanol tolerance, maintaining stable performance in the presence of methanol, unlike platinum catalysts which show significant voltage shifts due to methanol oxidation at the cathode. This methanol tolerance is particularly important for hybrid water electrolyzer applications where both electrodes are exposed to the methanol-containing electrolyte. When integrated into a hybrid anion exchange membrane water electrolyzer, the c-CMO catalyst achieved a current density of 1 A/cm^2^ at only 2 V and 60 °C, significantly outperforming conventional water electrolyzers that require 2.25 V or higher to achieve similar current densities.

The superior performance of the c-CMO catalyst is attributed to several factors including the unique capsule-like morphology that provides high surface area and abundant active sites, the synergistic interaction between copper and molybdenum that optimizes electronic structure and binding energies for reaction intermediates, and the good electrical conductivity of the porous nickel foam support that facilitates charge transfer. The strong Mo-Cu interaction modifies the electronic properties of both metals, enhancing their catalytic activity beyond what would be expected from a simple physical mixture. This case study demonstrates the potential of rationally designed transition metal-based catalysts for practical hybrid water splitting applications and highlights the importance of morphology control and compositional optimization in catalyst development [27,32,33,34].

### 5.3. 2D–2D NiMo-LDH/MXene Hybrid Electrocatalyst for Efficient Water Splitting

**Figure 19:**
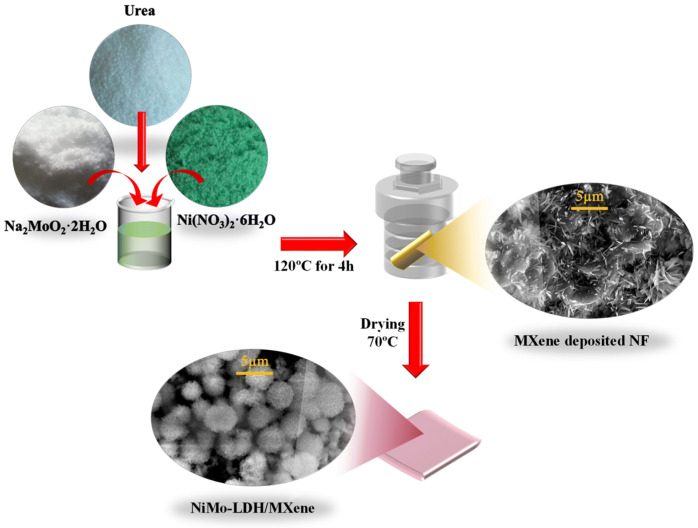
Schematic of the hydrothermal synthesis of the NiMo-LDH/MXene hybrid electrocatalyst. Reprinted with permission from [89].

**Figure 20:**
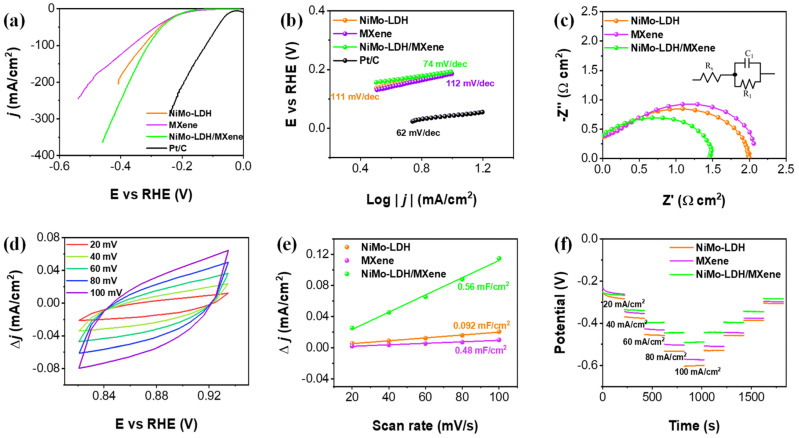
Hydrogen evolution reaction of NiMo-LDH, MXene, and NiMo-LDH/MXene showing (**a**) linear sweep voltammetry curves; (**b**) Tafel slopes; (**c**) Nyquist plots of NiMo-LDH, MXene, and NiMo-LDH/MXene, with Rs values of 2.7 U cm^2^, 1.9 U cm^2^ and 1.5 U cm^2^, respectively; (**d**) cyclic voltammetry curves of NiMo-LDH/MXene recorded at different scan rates; (**e**) linear plots of the capacitive current density vs. scan rate; (**f**) step chronopotentiometry at different current densities. Reprinted with permission from [89].

Samruddhi V. Chauhan et al. [89] designed and fabricated hierarchical 2D–2D hybrid electrocatalyst composed of NiMo-layered double hydroxide (NiMo-LDH) nanoflowers integrated with exfoliated MXene nanosheets on a porous nickel foam substrate for efficient overall water splitting (Figure 19). The synthesis involves etching Ti_3_AlC_2_ to obtain Ti_3_C_2_Tx MXene, followed by deposition onto nickel foam and subsequent hydrothermal growth of NiMo-LDH nanoflowers. The resulting architecture prevents restacking of MXene layers and exposes abundant sites. Structural and compositional analyses (XRD, SEM, TEM, XPS) confirm uniform integration of NiMo-LDH within MXene sheets, enhanced interfacial coupling, and optimized electronic structure, which collectively facilitate efficient charge transfer and catalytic activity. Electrochemical evaluation in alkaline media demonstrates excellent bifunctional performance (Figure 20). The hybrid catalyst achieves low overpotentials of 266 mV (HER) and 290 mV (OER) at 50 mA cm^−2^, outperforming individual components. At higher current densities (200 mA cm^−2^), it maintains competitive performance, indicating suitability for industrial applications. The improved activity is attributed to increased electrochemically active surface area, reduced charge transfer resistance, and synergistic interaction between NiMo-LDH (active sites) and MXene (conductive pathways). Mechanistically, Ni active sites facilitate water dissociation and redox transitions (Ni^2+^/Ni^3+^), while Mo enhances electronic conductivity and hydrogen adsorption/desorption kinetics. MXene contributes surface functional groups (–O, –OH, –F) that improve water adsorption and electron transport. The catalyst also exhibits strong durability, maintaining stable operation for ~90 h at high current densities (300–1000 mA cm^−2^) with high Faradaic efficiencies (~94% HER, ~80% OER). Overall, this work highlights that rational integration of LDH and 2D conductive materials offers a scalable strategy for high-performance, durable electrocatalysts for large-scale hydrogen production via water electrolysis. Table 1 and Table 2 compares the key parameters and electrochemical properties of different electrocatalysts used in water splitting reaction.

## 6. Conclusions and Future Perspectives

The development of efficient and cost-effective electrocatalysts for water splitting and hybrid water splitting represents a critical challenge for enabling large-scale sustainable hydrogen production. Significant progress has been made in understanding the fundamental mechanisms of HER and OER, developing advanced characterization techniques to probe catalyst structure and composition under operating conditions, and designing diverse catalyst materials ranging from noble metals to earth-abundant transition metals and metal-free carbons. State-of-the-art catalysts now achieve overpotentials as low as 10–30 mV for HER and 200–300 mV for OER at 10 mA cm^−2^, with some systems sustaining >100–1000 h stability. However, achieving the simultaneous combination of high activity, long-term durability, and low cost at industrial current densities (≥500–1000 mA cm^−2^) remains challenging.

Noble metal catalysts still define performance benchmarks, with Pt exhibiting near-zero overpotential for HER and IrO_2_ requiring ~200–300 mV at 10 mA cm^−2^ in acidic media. However, their scarcity (e.g., Pt and Ir < 0.001 ppm in Earth’s crust) and high cost (>$30–60 g^−1^) necessitate reduced loadings (<0.05 mg cm^−2^) or replacement. Transition metal-based catalysts (Ni, Co, Fe) show strong promise in alkaline systems, often achieving HER overpotentials of 50–150 mV and OER overpotentials of 250–350 mV, though stability in acidic media (<100 h in many cases) and activity gaps relative to noble metals persist. Metal-free carbon-based catalysts represent an exciting frontier that could eliminate dependence on metal resources, but typically require >300 mV overpotential and suffer from limited durability.

Hybrid water splitting systems offer a promising pathway to improved energy efficiency by replacing OER (thermodynamic potential 1.23 V) with more favorable oxidation reactions (often 0.2–0.8 V lower), reducing overall cell voltages to ~1.2–1.6 V at 10 mA cm^−2^. The integration of hydrogen production with organic oxidation, biomass valorization, or wastewater treatment creates synergies that enhance both the economic viability and environmental benefits of electrolytic hydrogen production. However, practical deployment requires catalysts with >90% selectivity, long-term stability (>500 h), and optimized system design, alongside careful techno-economic analysis.

Future research directions should focus on several key areas to advance the field toward practical applications. First, the development of operando and in situ characterization capabilities to observe catalysts under actual operating conditions will provide crucial insights into active site structures, reaction mechanisms, and degradation pathways that can guide rational catalyst design. Second, computational modeling and machine learning approaches can accelerate catalyst discovery by predicting promising compositions and structures for experimental validation. Third, the development of acid-stable non-noble metal catalysts capable of sustaining >500 mA cm^−2^ for >1000 h is critical for PEM electrolyzers. Fourth, scaling up catalyst synthesis methods from laboratory to industrial scale while maintaining performance and reducing costs to <$10 kW^−1^ is essential for commercialization.

Additionally, research into novel catalyst architectures such as single-atom catalysts, high-entropy alloys, and hierarchically structured materials may lead to breakthroughs in performance. The integration of catalysts with advanced electrode designs, membrane materials, and system engineering approaches will be necessary to translate catalyst advances into practical devices. Economic and life cycle analyses should accompany technical development to ensure that new technologies are truly sustainable and cost-effective (targeting hydrogen production costs below $2–3 kg^−1^) when considering the entire value chain from raw materials to end-of-life disposal or recycling. By addressing these challenges through coordinated efforts in fundamental research, applied development, and system integration, the vision of abundant clean hydrogen produced through efficient water splitting can be realized, contributing significantly to global decarbonization and sustainable energy goals.

## Figures and Tables

**Table 1:** Comparison of key parameters of the materials used for water splitting.

Catalyst Type	Representative Materials	Key Reactions (HER/OER/Hybrid)	Typical Performance	Electrolyte Compatibility	Key Design Strategies	Refs.
Noble Metal-Based Catalysts	Pt, IrO_2_, RuO_2_	HER (Pt), OER (IrO_2_, RuO_2_)	HER: ~0 mVOER: low overpotential (~300 mV)	Acidic & alkaline	Alloying, core–shell, nanostructuring	[12,13,14,15,40,41,42,43]
Transition Metal-Based Catalysts	Ni, Co, Fe, Mo compounds	HER & OER	HER: 100–300 mVOER: 250–400 mV	Mainly alkaline	Doping, heterostructures, reconstruction	[38,39,40,41,42,43,44,63]
Nickel-Based Catalysts	Ni(OH)_2_, NiOOH, NiFe-LDH	OER (dominant), HER	Comparable to IrO_2_ (alkaline OER)	Alkaline	Fe doping, LDH design, NiOOH formation	[44]
Cobalt-Based Catalysts	Co_3_O_4_, CoP, CoS	HER & OER	Moderate HER/OER	Alkaline	Mixed-metal tuning, phase transformation	[45,46,47,48,49,50,51]
Molybdenum-Based Catalysts	MoS_2_, Mo_2_C	HER	HER: ~150–300 mV	Acidic & alkaline	Exfoliation, vacancies, carbon hybridization	[52,53,54,55,56,57,58]
Copper-Based Catalysts	Cu-Mo, Cu_2_MnS_2_	HER + Hybrid reactions	HER: ~130 mV (hybrid)	Alkaline	Bimetallic synergy, plasma modification	[59,60,61,62,63]
Metal-Free Carbon Catalysts	Graphene, CNTs, g-C_3_N_4_	HER & OER (limited)	High overpotential (>300 mV)	Alkaline	Heteroatom doping (N, P, S), defects	[64,65,66,67,68,69,70,71,72,73]
Bifunctional Catalysts	NiFe-LDH, FeCoNi alloys	HER + OER	Cell voltage: ~1.6–2.0 V	Alkaline	Multi-metal synergy, interface design	[20,21,22,23,38,39,40,41,42,43,44]
Hybrid Water Splitting Catalysts	Cu-Mo oxide, Ni-based (urea)	HER + organic oxidation	Voltage ↓ by ~0.2–0.5 V	Alkaline	Coupled oxidation reactions	[74,75,76,77,78,79,80,81]
High-Entropy/Advanced Catalysts	Multi-metal alloys (HEPs)	HER & OER	Enhanced durability & kinetics	Alkaline	Entropy engineering, atomic tuning	[9,40,41,42,43]

**Table 2:** Performance comparison of electrocatalysts for water splitting.

Representative Materials	Overpotential (η)	Tafel Slope (mV dec^−1^)	Current Density (Benchmark)	Stability (Long-Term)	Mass Activity/TOF	Electrolyte	Key Design Strategy	Refs.
Pt, IrO_2_, RuO_2_	HER: ~0–50 mV; OER: ~200–300 mV	30–60	10 mA cm^−2^ (std), up to 1000 mA cm^−2^	>500–1000 h	High (benchmark; Pt ~1–10 s^−1^ TOF)	Acidic & alkaline	Alloying, nanostructuring	[12,13,14,15,40,41,42,43]
Ni, Co, Fe, Mo compounds	HER: 100–300 mV; OER: 250–400 mV	40–120	10–500 mA cm^−2^	50–500 h	Moderate	Alkaline	Doping, heterostructures	[38,39,40,41,42,43,44,63]
Ni(OH)_2_, NiOOH, NiFe-LDH	OER: ~250–350 mV	30–80	10–100 mA cm^−2^	>300 h	Moderate–high	Alkaline	Fe doping, LDH tuning	[44]
Co_3_O_4_, CoP, CoS	200–350 mV	50–120	10–200 mA cm^−2^	100–300 h	Moderate	Alkaline	Phase transformation	[45,46,47,48,49,50,51]
MoS_2_, Mo_2_C	150–300 mV	50–100	10–100 mA cm^−2^	100–200 h	Moderate	Acidic & alkaline	Defects, edge-site engineering	[52,53,54,55,56,57,58]
Cu-Mo, Cu_2_MnS_2_	~130 mV (HER)	~70–90	10–100 mA cm^−2^	~100 h	TOF reported (moderate-high)	Alkaline	Bimetallic synergy	[59,60,61,62,63]
Graphene, CNTs, g-C_3_N_4_	>300 mV	80–150	10–50 mA cm^−2^	<200 h	Low	Alkaline	Heteroatom doping	[64,65,66,67,68,69,70,71,72,73]
NiFe-LDH, FeCoNi alloys	Cell: ~1.6–2.0 V	40–100	10–500 mA cm^−2^	>300–800 h	Moderate–high	Alkaline	Interface engineering	[20,21,22,23,38,39,40,41,42,43,44]
Cu-Mo oxide, Ni-based (urea)	Reduced by 0.2–0.5 V vs. OER	40–90	10–1000 mA cm^−2^	~100–500 h	High (TOF up to ~1–5 s^−1^)	Alkaline	Coupled reactions	[74,75,76,77,78,79,80,81]
Multi-metal alloys	200–350 mV	40–80	10–500 mA cm^−2^	>500 h	High	Alkaline	Entropy engineering	[9,40,41,42,43]

## Data Availability

No new data was created or analyzed in this study.
